# Layer-Dependent Sensing Performance of WS_2_-Based Gas Sensors

**DOI:** 10.3390/nano14020235

**Published:** 2024-01-22

**Authors:** You Zhou, Sheng Wang, Sichen Xin, Sezin Sayin, Zhiqiang Yi, Zhenyu Li, Mona Zaghloul

**Affiliations:** 1 Department of Electrical & Computer Engineering, The George Washington University, 800 22nd Street, Washington, DC 20052, USA; sxin51@gwu.edu (S.X.); zhiqiang.yi@gwu.edu (Z.Y.); 2 Department of Biomedical Engineering, The George Washington University, 800 22nd Street, Washington, DC 20052, USAzhenyu@gwu.edu (Z.L.)

**Keywords:** 2D material, tungsten disulfide (WS_2_), layer engineering, gas sensor, gas monitoring system

## Abstract

Two-dimensional (2D) materials, such as tungsten disulfide (WS_2_), have attracted considerable attention for their potential in gas sensing applications, primarily due to their distinctive electrical properties and layer-dependent characteristics. This research explores the impact of the number of WS_2_ layers on the ability to detect gases by examining the layer-dependent sensing performance of WS_2_-based gas sensors. We fabricated gas sensors based on WS_2_ in both monolayer and multilayer configurations and methodically evaluated their response to various gases, including NO_2_, CO, NH_3_, and CH_4_ at room temperature and 50 degrees Celsius. In contrast to the monolayer counterpart, the multilayer WS_2_ sensor exhibits enhanced gas sensing performance at higher temperatures. Furthermore, a comprehensive gas monitoring system was constructed employing these WS_2_-based sensors, integrated with additional electronic components. To facilitate user access to data and receive alerts, sensor data were transmitted to a cloud-based platform for processing and storage. This investigation not only advances our understanding of 2D WS_2_-based gas sensors but also underscores the importance of layer engineering in tailoring their sensing capabilities for diverse applications. Additionally, the development of a gas monitoring system employing 2D WS_2_ within this study holds significant promise for future implementation in intelligent, efficient, and cost-effective sensor technologies.

## 1. Introduction

Carbon monoxide (CO), nitrogen dioxide (NO_2_), methane (CH_4_), and ammonia (NH_3_) are all types of air pollution [1,2,3]. Methane is also a typical gas that can cause a greenhouse effect [4,5]. Due to their potential to induce respiratory and cardiovascular issues, these gases are hazardous to both the environment and human health [1,6,7]. As reported by Philip J. Landrigan et al., 16% of all fatalities globally are caused by air pollution, which is the leading global cause of sickness and early death [8]. Thus, a sensitive, reliable, cost-effective, and easy-to-use gas monitoring system remains in high demand.

Traditional chemiresistor-type sensors are based on metal oxide film. These sensors typically have high sensitivity to gases, but they are difficult to handle and require a high operating temperature (200 °C to 500 °C) [9,10,11]. With time, two-dimensional transition metal dichalcogenides (TMDC)-based chemiresistor-type gas sensors have become increasingly important for spotting explosive, flammable, and poisonous gases in indoor and outdoor environments [12,13]. TMDC materials are often defined by the formula MX_2_, where M stands for a transition metal (Mo, W, Nb, Ti, or Ta) and X for a chalcogenide (S, Se, or Te). The TMDC materials usually have remarkable electrical, optical, and mechanical properties when they are fabricated as single or multiple layers [14,15,16,17,18,19,20,21,22]. The most extensively studied TMDC at the moment are MoS_2_ and WS_2_. MoS_2_ has a direct bandgap of 1.88 eV as a monolayer and indirect bandgap of 1.23 eV as bulk material, while WS_2_ has a 2.03 eV direct bandgap as a monolayer and 1.32 eV indirect bandgap as bulk [23]. Thus, they are prospective candidates for optoelectronic devices because of this feature [24]. The gas sensing performance of the 2D materials will change as the layer number or thickness changes because of their different properties (bandgap, conductance, gas adsorption rate, etc.). For example, the recovery time of the 2D-material-based ammonia gas sensor can be sped up by reducing the layer number Ref. [11]. Shumao Cui et al. reported that the relationship between sensitivity and the thickness of the phosphorene nanosheets (PNS) is governed by the band gap in thinner sheets (less than 10 nm) and by the effective thickness’s influence on gas adsorption in thicker sheets (greater than 10 nm) [25]. Compared to most metal oxide chemiresistor sensors, the 2D material gas sensors offer a significant advantage of enhanced sensitivity (for example, our 2D material sensor can detect NO_2_ at 200 ppb) due to their high surface-area-to-volume ratio. The thinness and unique electronic properties of 2D materials allow for operations with minimal energy requirements, which is advantageous for portable and wearable sensors [15,20]. In addition, the 2D material gas sensor can operate effectively at lower temperatures compared to metal oxide gas sensors, reducing energy consumption and expanding application possibilities. Furthermore, 2D-material-based gas sensors have low electrical noise, which makes them very suitable to detect gases at low concentrations with crucial response signals [26].

The sensing mechanism of 2D MoS_2_ and WS_2_ depends on a gas-induced doping effect [27,28]. The sensing materials may donate or take charges depending on the type of gases (oxidizing or reducing), which causes subsequent changes in sensor conductance in accordance, allowing the sensor to detect the presence of gas molecules [29,30,31]. The sensing materials, on the other hand, may revert to their initial condition again when the sensor is exposed to air or pure nitrogen due to the desorption of gas molecules. Strong or weak interactions may develop due to variations in the adsorption energies of different types of adsorbates and absorbents. Theoretical research on the adsorption energy between a test gas molecule and a mono- or multilayer WS_2_ found that polar gas molecules, such as NH_3_ and NO_2_, had higher adsorption energy than non-polar or weak polar ones, such as O_2_, CH_4_, and CO [15]. In addition, high adsorption energy typically affects gas sensing performance because the gas molecules will bind more strongly to the sensor, which potentially increases sensitivity as more molecules are detected [32,33]. However, stronger adsorption energy may lead to slower recovery or desorption rates, which is not ideal in applications where rapid detection and clearance are critical. In summary, both the binding energy and the electron transfer will affect the performance of 2D material gas sensors [11].

Comparatively little research has been conducted on the potential use of 2D WS_2_ for chemical gas sensors as opposed to MoS_2_. In this work, we tested the gas sensing performance of chemical vapor deposition (CVD)-grown monolayer and multilayer WS_2_ sensors under different temperatures and using different target gases. We found that the multilayer WS_2_ sensor showed higher sensitivity towards the test gases than the monolayer one. However, the multilayer sensor took more time to recover after the gases were removed. In addition, the sensing ability of the sensors can be enhanced at high temperatures. These findings underscore the potential of layer engineering in tailoring the sensing properties of two-dimensional materials to be used in different applications and meet specific environmental requirements. We also observed that the WS_2_ sensors exhibited longer recovery time when exposed to ammonia, while demonstrating higher sensitivity to NO_2_. The underlying reason is that NO_2_, acting as an oxidizing agent, exhibits high efficiency in accepting electrons from the surface of WS_2_ sensors. This electron transfer results in a more significant modification of the electrical properties of WS_2_. Although ammonia also engages in charge transfer interactions, its effect on the electronic properties of WS_2_ is comparatively less dramatic, resulting in lower sensitivity compared to NO_2_. The molecular structure and polarity of NH_3_ facilitate the formation of robust dipole interactions with the WS_2_ surface. In contrast, despite NO_2_’s efficacy as a strong oxidizing agent, it may not establish interactions of similar polar strength with WS_2_ as observed with NH_3_. Thus, the WS_2_ sensors need more time to recover after exposure to NH_3_ because of this strong binding. We tested the multilayer WS_2_ sensor with low-concentration NO_2_ gas and compared its performance with other commercial gas sensors. We can conclude that the primary technical advantage of the multilayer WS_2_ gas sensor is its balanced performance profile. It provides an effective combination of measurement range, sensitivity (limit of detection, LOD), and response time. This balance renders the sensor potentially more adaptable for a wide range of applications, particularly where rapid detection of low concentrations of NO_2_ is critical. Its LOD and response time are suited for environments where moderate fluctuations in NO_2_ levels are expected and require monitoring with a considerable degree of accuracy and speed. The multilayer WS_2_ sensor was finally integrated with some electronic components to build a gas monitoring system. The data collected from the sensors were uploaded to the cloud for real-time monitoring. This gas monitoring system is light, sensitive, cost-effective, efficient, and easy to use.

## 2. Materials and Methods

### 2.1. Sample Preparation and Characterization

**Figure 1:**
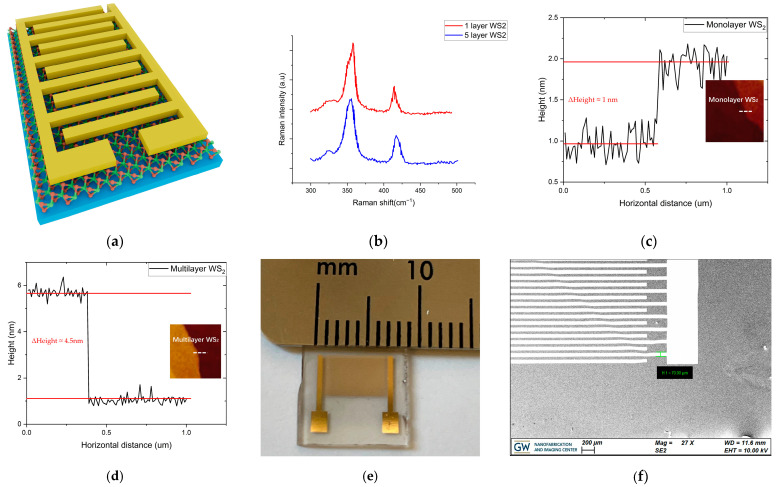
(**a**) Planar structure of the WS_2_-based gas sensing device; (**b**) Raman spectra of 1-layer and 5-layer WS_2_; (**c**) AFM measurement for monolayer WS_2_; (**d**) AFM measurement for multilayer WS_2_; (**e**) Optical photograph of the monolayer WS_2_ gas sensing device; (**f**) SEM image of the comb-structural electrodes.

The monolayer and multilayer WS_2_ material was grown on sapphire substrate. The multilayer WS_2_ sample has identical structure and size to the monolayer sample. Figure 1a illustrates the planar structure and schematic of the 2D WS_2_ sensor in this work. The most common structure configuration of 2D WS_2_ gas sensor is planar structure because it is easy to develop and manufacture and has potential cost savings [34,35,36]. Figure 1b presents the Raman spectra of WS_2_ samples with 1 layer and multiple layers that were used in this work. The excitation wavelength is 514 nm. The frequency difference between the E^1^_2g_ mode and A_1g_ mode of monolayer WS_2_ is around 65 cm^−1^, while frequency difference of multilayer WS_2_ is about 70 cm^−1^. The measured frequency difference of the WS_2_ samples in this work coincides well with the measurement from other reports for WS_2_ on sapphire [37,38]. To further identify the layer number, the thickness of the WS_2_ samples was measured by Atomic Force Microscope (AFM). The thickness measured by AFM (Bruker, Camarillo, CA, USA. Innova type, soft tapping mode, RFESPA-40 probe, and 40 KHz frequency) for monolayer WS_2_ is about 1 nm, while the thickness of multilayer WS_2_ is about 4.5 nm, which is consistent with the reported thickness of 1 and 5 layers WS_2_ as shown in Figure 1c,d [11]. Figure 1e shows an optical photograph of the monolayer WS_2_ gas sensing device used in this work. Multilayer WS_2_ device has identical structure and size. Figure 1f shows SEM image of the comb-structural electrodes. The width of electrodes is 70 um and the length is 6 mm.

**Figure 2:**
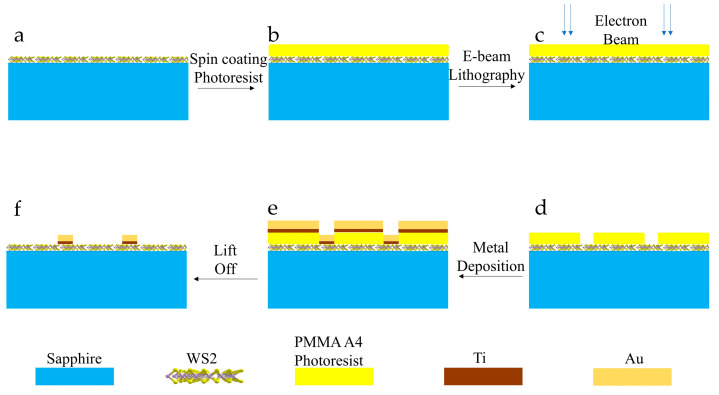
Fabrication process of the WS_2_ sensor devices. (**a**) 2D WS_2_ fabricated on a clean sapphire substrate by CVD; (**b**) Photoresist deposition; (**c**) EBL-lithography; (**d**) Development after lithography; (**e**) Metal deposition; (**f**) Lift off.

Comb-structural electrodes were fabricated on top of the WS_2_ sensing layer as shown in Figure 2. The fabrication process illustrated in Figure 2 is as follows: a. the monolayer or multilayer WS_2_ was grown on clean sapphire substrate by chemical vapor deposition(CVD). b. 600 nm PMMA A4 photoresist was deposited on top of the 2D WS_2_ by spin coating. Then, baked the sample at 180 °C for 2 min after coating. c. Raith Electron Beam Lithography (EBL) system was used for patterning the structure of the electrodes. d. Next step was to develop the post-lithography sample using methyl isobutyl ketone (MIBK)/isopropyl alcohol (IPA): 1/3 positive photoresist developer for 1 min to remove part of photoresist exposed to electron beam in lithography step. e. 5 nm Ti and 200 nm Au were deposited on the wafer using CHA criterion electron beam evaporator. Titanium was used as an adhesive layer. f. The photoresist and the metal on it were removed by immersing the sample in acetone for 30 min. After that, the comb-structural electrodes were fabricated on the sample.

### 2.2. Experimental Setup

**Figure 3:**
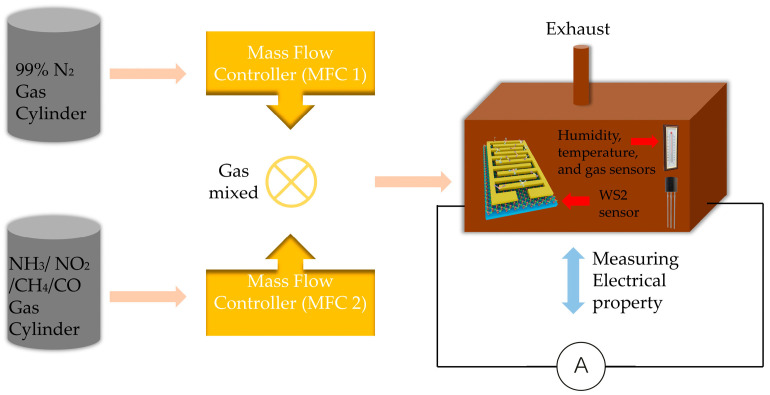
Experimental setup used for gas sensing performance test.

The experimental setup is shown in Figure 3. The monolayer and multilayer WS_2_ gas sensors were tested with different gases at room temperature and 50 °C. The mass flow controller 1 (MFC 1) controlled the flow rate of nitrogen, while MFC 2 controlled the flow rate of target gases (NH_3_, NO_2_, CH_4_, and CO). The mixed gas (diluting gas from MFC1 and target gas from MFC 2) was induced to the analytical chamber. In this work, we tested our samples with 100 ppm NH_3_, 500 ppm CH_4_, 500 ppm CO, and 1 ppm NO_2_. We also put temperature sensor, humidity sensor, and commercial gas sensors (electrochemical gas sensors from Dfrobot) inside the analytical chamber. The model of temperature sensor and humidity sensor is DHT 11. The relative humidity during the test was around 50–55%. The gas sensors were used for monitoring the gas concentration inside the analytical chamber when performing the tests. The electrical properties of the gas sensors were measured during the gas sensing test.

## 3. Results

### 3.1. Current–Voltage Characterization

**Figure 4:**
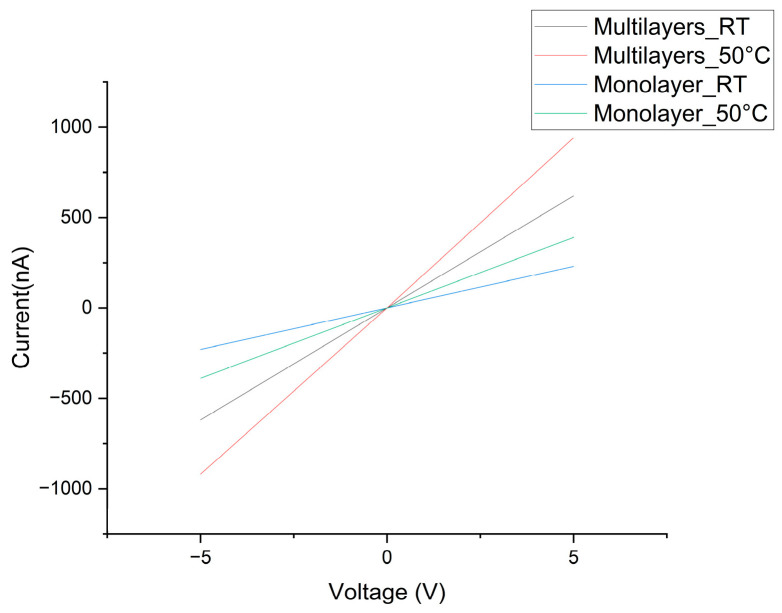
Current–voltage characteristics of monolayer and multilayer WS_2_ gas sensors measured under different temperatures.

Before conducting the gas sensing performance measurements, the electrical properties of the monolayer WS_2_ and multilayer WS_2_ samples were characterized at room temperature and 50 °C as shown in Figure 4. Multilayer WS_2_ has stronger conductance, and high temperature improves this property. All the curves reveal a linear relation between the measured current and the applied voltage, which ranges from −5 to +5 V.

### 3.2. Sensing Performance Tests

During the gas sensing test, the samples were always stabilized in nitrogen for 30 min before passing the test gases. The response of the WS_2_-based sensors upon testing with different gases is the change in current when applying 5 V on the sensors, as expressed in Equation (1), where $I_{0}$ is the measured current of the sensors when no analyte gas was injected into the chamber at room temperature (27 °C) or 50 °C and $I_{\mathrm{test}}$ is the measured current when the sensors were exposed to target gases.

(1) $$\mathrm{Response}=\left|\frac{I_{\mathrm{test}}{\;-\;I}_{0}}{I_{0}}\right|$$

**Figure 5:**
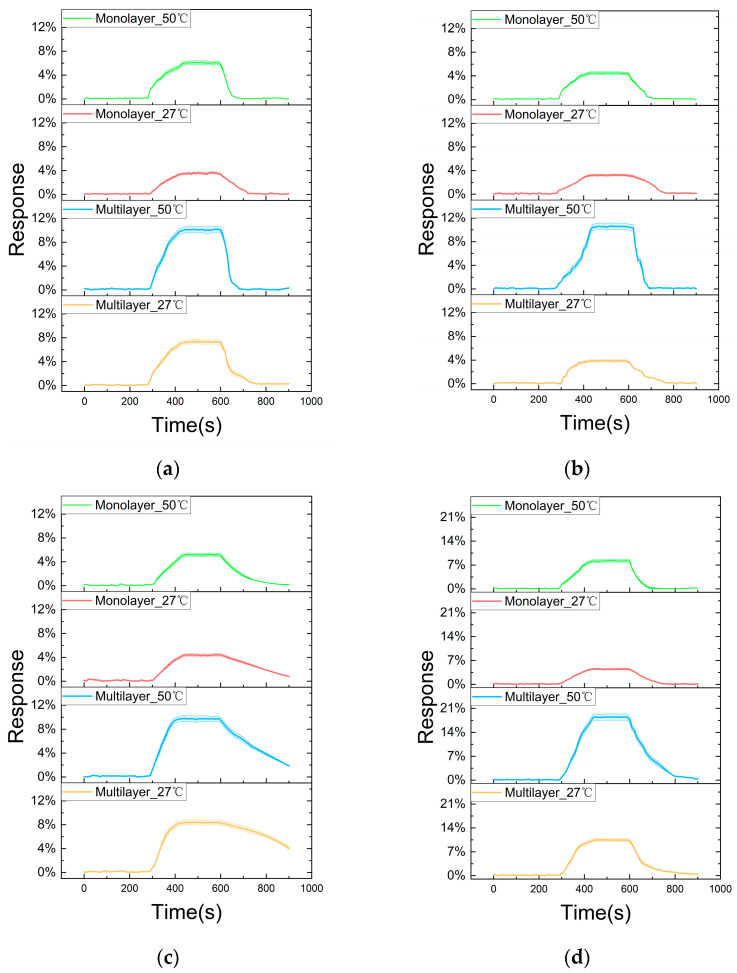
Comparative sensing behavior for mono- and multilayer WS_2_ at 27 °C and 50 °C: (**a**) 500 ppm CH_4_; (**b**) 500 ppm CO; (**c**) 100 ppm NH_3_; (**d**) 1 ppm NO_2_.

The monolayer and multilayer WS_2_ sensors were tested with different gases at room temperature (27 °C) and 50 °C. The analyte gas was injected into the chamber at 300 s and stopped at 600 s. Then, the samples were allowed to recover in pure nitrogen gas. The whole experiment lasted 900 s. We repeated the experiments four times and collected the average response data and measurement deviation, as shown in Figure 5.

Figure 5a shows the gas sensing behavior at different configurations when exposed to 500 ppm CH_4_ gas. The solid line presents average response data from the repeated experiments, while the shadow presents the deviation. Upon introduction of the gas, there is an immediate rise in response, which then stabilizes slightly. The steepest rise is observed for the multilayer sensor at 50 °C, and we can find maximum sensitivity in this configuration. Both sensor configurations, monolayer and multilayer, are more responsive at 50 °C than at room temperature. This reinforces the observation that the sensor is more sensitive or reactive at higher temperatures. In addition, at both temperatures, the multilayer configuration exhibits a more pronounced response than monolayer, which indicates that multilayer enhances the sensor’s ability to interact with gases. Moreover, after stopping the gas flow at 600 s, all the sensors begin to return to their baseline, with the multilayer sensor at 50 °C taking a longer time, indicating a stronger interaction or adsorption of CH_4_ molecules. Figure 5b shows the measurements of the response of gas sensors when exposed to 500 ppm CO. The result is similar to the test with CH_4_ except that the layer does not have a significant effect on the sensitivity at room temperature. Figure 5c displays the response of WS_2_ gas sensors when exposed to 100 ppm NH_3_. Interestingly, we can see that it takes a much longer time for the sensors to recover. Both higher temperature and increased layer number can enhance sensitivity. However, the layer number can exert more influence on the recovery rate when exposed to NH_3_. Since the WS_2_ sensors are more sensitive to NO_2_, we used 1 ppm NO_2_ for the test, as shown in Figure 5d. The WS_2_ gas sensor’s response to NO_2_ is notably affected by both its layer number and the temperature. The maximum sensitivity in this work was achieved by testing multilayer WS_2_ with 1 ppm NO_2_ at 50 °C.

**Figure 6:**
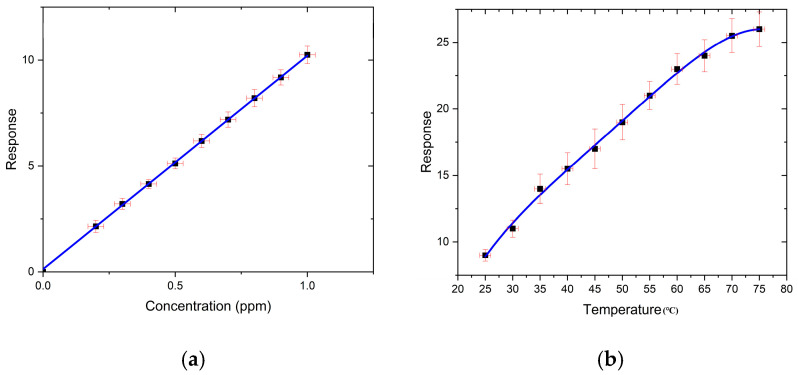
(**a**) Response to NO_2_ as a function of concentration for multilayer WS_2_ at 27 °C. (**b**) Response to 1 ppm NO_2_ at different temperatures.

For the multilayer WS_2_ gas sensor at room temperature, the limit of detection (LOD) values for CH_4_, CO, NH_3_, and NO_2_ are 130 ppm, 200 ppm, 25 ppm, and 0.2 ppm, respectively. From the above results, we can know that the multilayer WS_2_ sensor has good sensitivity to low-concentration NO_2_ even at room temperature. Thus, we tested this sensor with NO_2_ from 0.2 ppm to 1 ppm and found that the response is linear at room temperature, as shown in Figure 6a. We also tested the multilayer WS_2_ sensor with 1 ppm NO_2_ at different temperatures and found that the sensitivity increases as the temperature increases, as shown in Figure 6b.

Table 1 shows the comparison between the multilayer WS_2_ gas sensor and some commercial NO_2_ gas sensors [39,40,41]. The multilayer WS_2_ sensor has a measurement range of 0–10 ppm, which is wider than the Allsensing Gas Sensor (0–5 ppm) but narrower than the SGX and FIGARO sensors. This mid-range capability makes it suitable for applications where moderate or low concentration detection is required. The comparison of the limit of detection indicates that the multilayer WS_2_ sensor is quite sensitive, capable of detecting relatively low concentrations of NO_2_. The response time of the multilayer WS_2_ sensor is 95 s, which is significantly faster than the Allsensing sensor (150 s) but slower than the SGX (30 s) and FIGARO (25 s) sensors. This suggests that, while the multilayer WS_2_ sensor may not be the fastest in terms of response, it still offers a reasonable response time, making it suitable for applications where a balance between speed and sensitivity is needed.

In summary, the technical advantage of the multilayer WS_2_ gas sensor lies in its balanced performance. It offers a good combination of measurement range, sensitivity (LOD), and response time. This makes it potentially more versatile for various applications, especially when detecting low concentrations of NO_2_ quickly is necessary. Its LOD and response time make it suitable for environments where moderately small changes in NO_2_ levels are expected and need to be monitored with reasonable accuracy and speed.

### 3.3. Gas Monitoring System

**Figure 7:**
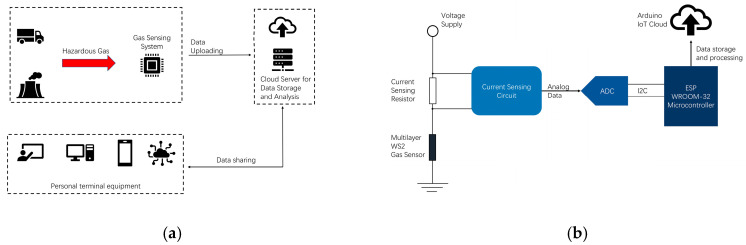
(**a**) Architecture of the gas monitoring system; (**b**) schematic of the gas sensing circuit.

Since the multilayer WS_2_ is able to detect low-concentration NO_2_ at room temperature, we developed a gas monitoring system based on this sensor device. This system is able to detect the concentration of target gas that is produced from a source and send the sensing data to the cloud server. The data will be processed and stored in the cloud server, from which people can access the data using their personal terminal equipment as shown in Figure 7a. Figure 7b delineates the schematic of the electronic system incorporating the gas sensor module. A current sensing circuit is employed to quantify the current traversing the WS_2_ gas sensor. The Analog-to-Digital Converter (ADC) captures the analog metrics from the current detection circuit and conveys the processed data to the ESP-WROOM-32 microcontroller. Subsequently, the microcontroller transmits the data to the Arduino IoT cloud platform.

**Figure 8:**
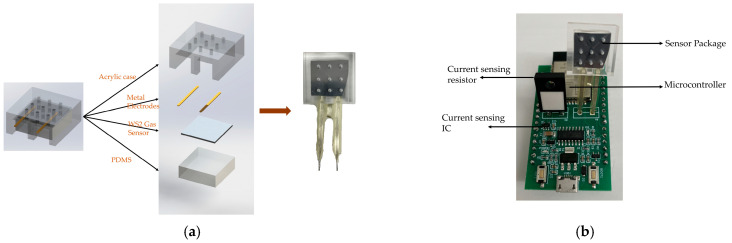
(**a**) Package of the WS_2_ gas sensor; (**b**) electronic components of the gas sensing system.

The circuit was built based on the above schematic. The WS_2_ gas sensor was packaged with polydimethylsiloxane (PDMS) and transparent acrylic as shown in Figure 8a. Figure 8b illustrates the gas sensing circuit including all the electronic components.

**Figure 9:**
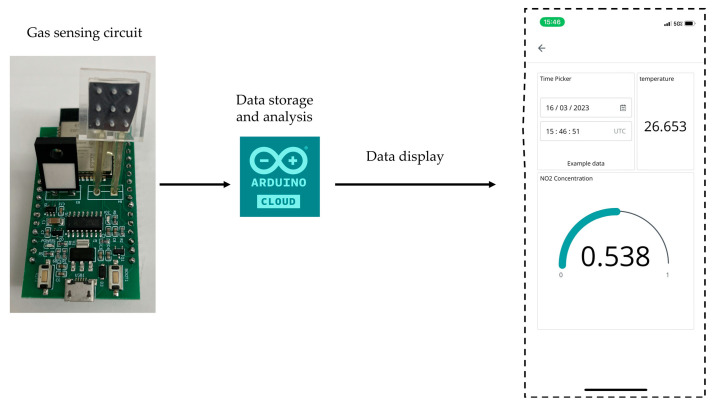
Data stored on cloud server and displayed on phone with GUI.

It is necessary to view the data stored on the cloud server so that users may monitor the presence and concentration of the gases and receive alerts. Thus, the sensing data were sent to Arduino IoT cloud for data storage and analysis. Then, we could access the data via phone. We designed a GUI to help people track changes in the concentration of NO_2_ gas easily. The information of concentration, time, and temperature can be accessed via phone as shown in Figure 9. The system may be utilized almost anywhere because it is lightweight and portable. To test the stability of this system, we tested this gas monitoring system with low concentrations of NO_2_ after several months and achieved the same performance.

## 4. Discussion

We observed that the WS_2_ gas sensors exhibited higher sensitivity towards NO_2_ and NH_3_ than CO and CH_4_. The reason is that NO_2_ and NH_3_ are polar molecules with higher adsorption energies compared to CO and CH_4_, which are weak polar and non-polar ones, meaning they bind more strongly to the WS_2_ surface. This stronger binding leads to a greater effect on the electrical properties of the sensor. We also found that the 2D WS_2_ gas sensors showed longer recovery time for NH_3_ but higher sensitivity to NO_2_. The recovery times when sensing NH_3_ for monolayer WS_2_ at 50 °C, monolayer WS_2_ at 27 °C, multilayer WS_2_ at 50 °C, and multilayer WS_2_ at 27 °C are 180 s, 320 s, 400 s, and 550 s, respectively. The recovery times when sensing NO_2_ for monolayer WS_2_ at 50 °C, monolayer WS_2_ at 27 °C, multilayer WS_2_ at 50 °C, and multilayer WS_2_ at 27 °C are 90 s, 120 s, 220 s, and 210 s, respectively. Sensitivity and recovery time are two distinct aspects of a gas sensor’s performance. Sensitivity refers to the ability of the sensor to detect low concentrations of a gas, often related to how the gas molecules interact with and change the electrical properties of the sensor. Recovery time, on the other hand, is about how quickly the sensor can return to its baseline state after stopping exposure to the gas. As an oxidizing agent, NO_2_ can effectively accept electrons from the WS_2_ surface, leading to a more significant change in its electrical properties. NH_3_, while it may also interact through charge transfer, does not induce as dramatic a change in the electronic properties of WS_2_, hence lower sensitivity than NO_2_. NH_3_’s structure and polarity enable it to establish stronger dipole interactions with the WS_2_ surface. In contrast, NO_2_, despite being a strong oxidizing agent, may not form as strong polar interactions with WS_2_ as NH_3_ does. In summary, the difference in interaction mechanisms with the WS_2_ sensor surface leads to these distinct behaviors. NO_2_, with its strong oxidizing nature, causes a significant and rapid change in the sensor’s electrical properties, leading to high sensitivity. In contrast, NH_3_’s stronger adsorption leads to a longer recovery time despite its lower sensitivity than NO_2_.

The WS_2_ gas sensor’s response to gases is notably affected by both its layer configuration and the temperature. The four-layer sensor at 50 °C demonstrates the strongest reaction to the gas introduction, indicating maximum sensitivity in this setup. This could suggest that, for applications where high sensitivity is required, a multilayered sensor operated at higher temperatures might be more effective. Conversely, the one-layer sensor at 27 °C might be more suited for environments where minimal sensitivity is needed or where energy conservation (via lower operating temperatures) is a priority.

## 5. Conclusions

We investigated the layer-dependent sensing capabilities of WS_2_-based gas sensors in this work, and we observed a strong correlation between the gas detection performance of these sensors and the number of WS_2_ layers. According to our findings, multilayer WS_2_ sensors provide improved gas sensing capabilities at high temperatures, whereas monolayer WS_2_ sensors also showed notable sensing properties. The sensitivity and recovery rate of the 2D material gas sensors exhibit variability when subjected to different gases. This variation is attributable to the influence of both the binding energy and the electron transfer processes on the sensors’ performance. The 2D material gas sensors function efficiently at different temperatures, leading to controllable energy usage and application in flammable and explosive environments. This discovery highlights how layer engineering may be used to customize 2D materials such as WS_2_’s sensing properties to meet a variety of applications and environment-specific needs.

Furthermore, the development of our gas monitoring system, which efficiently integrates WS_2_ sensors and cloud technology, paves the way for smart, reliable, and cost-effective sensing solutions. Compared to some commercial gas sensors, the foremost technical merit of the multilayer WS_2_ gas sensor is its balanced performance. It offers an effective combination of measurement range, sensitivity (limit of detection, LOD), and response time. Such advancements are not only vital for monitoring harmful pollutants and ensuring public health but also hold promise for many applications across various industries.

Even though this research has yielded important findings, there are still many unexplored possibilities to discover. Future research might concentrate on determining how WS_2_ sensors behave in various environmental settings, improving the integration of the electrical components, and determining if the suggested system is scalable for large-scale deployments. Our research paves the way for future developments in the field of gas sensing technology by highlighting the promising prospects of two-dimensional materials and the notable opportunities of complex layer engineering.

## Figures and Tables

**Table 1:** Comparison to some commercial gas sensors.

Gas Sensor	Measurement Range	Limit of Detection	Response Time (T90)
SGX Solid Polymer Electrolyte Gas Sensor (PS1-NO2-50)	0~50 ppm	1 ppm	30 s
FIGARO Solid Electrolyte Electrochemical Gas Sensor (FECS42-20)	0~20 ppm	Approximately 0.5 ppm	25 s
Allsensing Gas Sensor (AGSM-NO2-5)	0~5 ppm	0.1 ppm	150 s
Multilayer WS_2_ Gas Sensor	0~10 ppm	0.2 ppm	95 s

## Data Availability

The data that support the findings of this study are available from the corresponding author upon reasonable request.

## References

[1] Jung S.J., Mehta J.S., Tong L. (2018). Effects of Environment Pollution on the Ocular Surface. Ocul. Surf..

[2] Israt A. (2022). Analysis of Air Pollution Levels Due to Methane (Ch4) Emissions at Final Disposal Site. J. Asian Multicult. Res. Med. Health Sci. Study.

[3] Hasnain M.G., Garcia-Esperon C., Tomari Y.K., Walker R., Saluja T., Rahman M.M., Boyle A., Levi C.R., Naidu R., Filippelli G. (2023). Effect of Short-Term Exposure to Air Pollution on Daily Cardio- and Cerebrovascular Hospitalisations in Areas with a Low Level of Air Pollution. Env. Sci. Pollut. Res..

[4] Duval B.D., Martin J., Marsalis M.A. (2022). The Effect of Variable Fertilizer and Irrigation Treatments on Greenhouse Gas Fluxes from Aridland Sorghum. Agronomy.

[5] Fadlilah A., Jayadi M., Nathan M. (2023). Methane Gas Emissions (CH4) in Paddy Fields in Minasatene District, Pangkep Regency: Microbial Abundance against Increased Methane Gas. IOP Conf. Ser. Earth Environ. Sci..

[6] Bayer-Oglesby L., Grize L., Gassner M., Takken-Sahli K., Sennhauser F.H., Neu U., Schindler C., Braun-Fahrländer C. (2005). Decline of Ambient Air Pollution Levels and Improved Respiratory Health in Swiss Children. Environ. Health Perspect..

[7] Ho A.F.W., Hu Z., Woo T.Z.C., Tan K.B.K., Lim J.H., Woo M., Liu N., Morgan G.G., Ong M.E.H., Aik J. (2022). Ambient Air Quality and Emergency Hospital Admissions in Singapore: A Time-Series Analysis. Int. J. Environ. Res. Public Health.

[8] Landrigan P.J., Fuller R., Fisher S., Suk W.A., Sly P., Chiles T.C., Bose-O’Reilly S. (2019). Pollution and Children’s Health. Sci. Total Environ..

[9] Afzal A., Cioffi N., Sabbatini L., Torsi L. (2012). NOx Sensors Based on Semiconducting Metal Oxide Nanostructures: Progress and Perspectives. Sens. Actuators B Chem..

[10] Choi K.J., Jang H.W. (2010). One-Dimensional Oxide Nanostructures as Gas-Sensing Materials: Review and Issues. Sensors.

[11] Qin Z., Zeng D., Zhang J., Wu C., Wen Y., Shan B., Xie C. (2017). Effect of Layer Number on Recovery Rate of WS2 Nanosheets for Ammonia Detection at Room Temperature. Appl. Surf. Sci..

[12] Zhao S., Wang G., Liao J., Lv S., Zhu Z., Li Z. (2018). Vertically Aligned MoS_2_/ZnO Nanowires Nanostructures with Highly Enhanced NO_2_ Sensing Activities. Appl. Surf. Sci..

[13] Choi G.J., Mishra R.K., Gwag J.S. (2020). 2D Layered MoS_2_ Based Gas Sensor for Indoor Pollutant Formaldehyde Gas Sensing Applications. Mater. Lett..

[14] Yoon Y., Ganapathi K., Salahuddin S. (2011). How Good Can Monolayer MoS_2_ Transistors Be?. Nano Lett..

[15] Cao J., Chen Q., Wang X., Zhang Q., Yu H.-D., Huang X., Huang W. (2021). Recent Development of Gas Sensing Platforms Based on 2D Atomic Crystals. Research.

[16] Fiori G., Bonaccorso F., Iannaccone G., Palacios T., Neumaier D., Seabaugh A., Banerjee S.K., Colombo L. (2014). Electronics Based on Two-Dimensional Materials. Nat. Nanotechnol..

[17] Chhowalla M., Shin H.S., Eda G., Li L.-J., Loh K.P., Zhang H. (2013). The Chemistry of Two-Dimensional Layered Transition Metal Dichalcogenide Nanosheets. Nat. Chem..

[18] Dumcenco D., Ovchinnikov D., Marinov K., Lazić P., Gibertini M., Marzari N., Sanchez O.L., Kung Y.-C., Krasnozhon D., Chen M.-W. (2015). Large-Area Epitaxial Monolayer MoS_2_. ACS Nano.

[19] Manzeli S., Ovchinnikov D., Pasquier D., Yazyev O.V., Kis A. (2017). 2D Transition Metal Dichalcogenides. Nat. Rev. Mater..

[20] Wang B., Gu Y., Chen L., Ji L., Zhu H., Sun Q. (2022). Gas Sensing Devices Based on Two-Dimensional Materials: A Review. Nanotechnology.

[21] Wang Q.H., Kalantar-Zadeh K., Kis A., Coleman J.N., Strano M.S. (2012). Electronics and Optoelectronics of Two-Dimensional Transition Metal Dichalcogenides. Nat. Nanotechnol..

[22] Naumis G.G., Barraza-Lopez S., Oliva-Leyva M., Terrones H. (2017). Electronic and Optical Properties of Strained Graphene and Other Strained 2D Materials: A Review. Rep. Prog. Phys..

[23] Gusakova J., Wang X., Shiau L.L., Krivosheeva A., Shaposhnikov V., Borisenko V., Gusakov V., Tay B.K. (2017). Electronic Properties of Bulk and Monolayer TMDs: Theoretical Study Within DFT Framework (GVJ-2e Method). Phys. Status Solidi (a).

[24] Splendiani A., Sun L., Zhang Y., Li T., Kim J., Chim C.-Y., Galli G., Wang F. (2010). Emerging Photoluminescence in Monolayer MoS_2_. Nano Lett..

[25] Cui S., Pu H., Wells S.A., Wen Z., Mao S., Chang J., Hersam M.C., Chen J. (2015). Ultrahigh Sensitivity and Layer-Dependent Sensing Performance of Phosphorene-Based Gas Sensors. Nat. Commun..

[26] Varghese S.S., Varghese S.H., Swaminathan S., Singh K.K., Mittal V. (2015). Two-Dimensional Materials for Sensing: Graphene and Beyond. Electronics.

[27] Bag A., Lee N.-E. (2019). Gas Sensing with Heterostructures Based on Two-Dimensional Nanostructured Materials: A Review. J. Mater. Chem. C.

[28] Pham P.V., Bodepudi S.C., Shehzad K., Liu Y., Xu Y., Yu B., Duan X. (2022). 2D Heterostructures for Ubiquitous Electronics and Optoelectronics: Principles, Opportunities, and Challenges. Chem. Rev..

[29] Mathew M., Shinde P.V., Samal R., Rout C.S. (2021). A Review on Mechanisms and Recent Developments in P-n Heterojunctions of 2D Materials for Gas Sensing Applications. J. Mater. Sci..

[30] Bag A., Lee N.-E. (2021). Recent Advancements in Development of Wearable Gas Sensors. Adv. Mater. Technol..

[31] Mathew M., Sekhar Rout C. (2021). Schottky Diodes Based on 2D Materials for Environmental Gas Monitoring: A Review on Emerging Trends, Recent Developments and Future Perspectives. J. Mater. Chem. C.

[32] Kumar R., Goel N., Hojamberdiev M., Kumar M. (2020). Transition Metal Dichalcogenides-Based Flexible Gas Sensors. Sens. Actuators A Phys..

[33] Joshi N., Hayasaka T., Liu Y., Liu H., Oliveira O.N., Lin L. (2018). A Review on Chemiresistive Room Temperature Gas Sensors Based on Metal Oxide Nanostructures, Graphene and 2D Transition Metal Dichalcogenides. Microchim. Acta.

[34] Alene Asres G., Baldoví J.J., Dombovari A., Järvinen T., Lorite G.S., Mohl M., Shchukarev A., Pérez Paz A., Xian L., Mikkola J.-P. (2018). Ultrasensitive H2S Gas Sensors Based on P-Type WS2 Hybrid Materials. Nano Res..

[35] Bhattacharyya P., Acharyya D., Dutta K., Dasgupta N., Ranjan S., Lichtfouse E. (2019). Resistive and Capacitive Measurement of Nano-Structured Gas Sensors. Environmental Nanotechnology: Volume 2.

[36] Bhattacharyya P., Acharyya D. (2021). Impact of Device Configurations on Sensing Performance of WS_2_-Based Gas Sensors: A Review. IEEE Sens. J..

[37] Zeng H., Liu G.-B., Dai J., Yan Y., Zhu B., He R., Xie L., Xu S., Chen X., Yao W. (2013). Optical Signature of Symmetry Variations and Spin-Valley Coupling in Atomically Thin Tungsten Dichalcogenides. Sci. Rep..

[38] Zhang Y., Zhang Y., Ji Q., Ju J., Yuan H., Shi J., Gao T., Ma D., Liu M., Chen Y. (2013). Controlled Growth of High-Quality Monolayer WS2 Layers on Sapphire and Imaging Its Grain Boundary. ACS Nano.

[39] PS1-NO2-50 SGX Sensortech Gas Sensor—Solid Polymer Sensor|SGX. https://sgx.cdistore.com/products/detail/ps1no250-sgx-sensortech/741050/.

[40] FECS42-20: Gas Sensors & Modules—Products—Figaro Engineering Inc. https://www.figarosensor.com/product/entry/fecs42-20.html.

[41] AGSM-NO2-5. https://www.k-allsensing.com/products/agsm-no2-5.

